# Genomic profiles of renal cell carcinoma in a small Chinese cohort

**DOI:** 10.3389/fonc.2023.1095775

**Published:** 2023-06-22

**Authors:** Sheng Tai, Dan-dan Xu, Zhixian Yu, Yu Guan, Shuiping Yin, Jun Xiao, Song Xue, Chaozhao Liang

**Affiliations:** ^1^ Department of Urology, The First Affiliated Hospital of Anhui Medical University, Hefei, Anhui, China; ^2^ Institute of Urology, Anhui Medical University, Hefei, Anhui, China; ^3^ Anhui Province Key Laboratory of Genitourinary Diseases, Anhui Medical University, Hefei, Anhui, China; ^4^ Department of Oncology, Hospital of Anhui Medical University, Hefei, Anhui, China; ^5^ Department of Oncology, Anhui Public Health Clinical Center, Hefei, China; ^6^ Department of Urology, The First Affiliated Hospital of Wenzhou Medical University, Wenzhou, China; ^7^ Department of Urology, The First Affiliated Hospital of University of Science and Technology of China (USTC), Division of Life Sciences and Medicine, University of Science and Technology of China, Hefei, China; ^8^ Department of Urology, General Hospital of Eastern Theater Command, Nanjing, China

**Keywords:** renal cell carcinoma (RCC), clear cell renal cell carcinoma (ccRCC), non-clear cell renal cell carcinoma (nccRCC), mutation, VHL, FH

## Abstract

**Objectives:**

Our aim was to describe the molecular characteristics of Renal Cell Carcinoma (RCC) and develop a small panel of RCC-associated genes from a large panel of cancer-related genes.

**Materials and methods:**

Clinical data of 55 patients with RCC diagnosed in four hospitals from September 2021 to August 2022 were collected. Among the 55 patients, 38 were diagnosed with clear cell RCC (ccRCC), and the other 17 were diagnosed with non-clear cell RCC (nccRCC), including 10 cases of papillary renal cell carcinoma, 2 cases of hereditary leiomyomatosis and RCC syndrome (HLRCC), 1 eosinophilic papillary RCC, 1 tubular cystic carcinoma, 1 TFE3 gene fusion RCC, and 2 RCC with sarcomatoid differentiation. For each patient, 1123 cancer-related genes and 79 RCC-associated genes were analyzed.

**Results:**

The most frequent mutations in a large panel of 1123 cancer-related genes in the overall population of RCC patients were VHL (51%), PBRM1 (35%), BAP1 (16%), KMT2D (15%), PTPRD (15%), and SETD2 (15%). For ccRCC patients, mutations in VHL, PBRM1, BAP1, and SERD2 can reach 74%, 50%, 24%, and 18%, respectively, while for nccRCC patients, the most frequent mutation was FH (29%), MLH3 (24%), ARID1A (18%), KMT2D (18%), and CREBBP (18%). The germline mutation rate in all 55 patients reached 12.7% (five with FH, one with ATM, and one with RAD50). The small panel containing only 79 RCC-associated genes demonstrated that mutations of VHL, PBRM1, BAP1, and SETD2 in ccRCC patients were 74%, 50%, 24%, and 18% respectively, while for the nccRCC cohort, the most frequent mutations were FH (29%), ARID1A (18%), ATM (12%), MSH6 (12%), BRAF (12%), and KRAS (12%). For ccRCC patients, the spectrum of mutations by large and small panels was almost the same, while for nccRCC patients, the mutation spectrum showed some differences. Even though the most frequent mutations (FH and ARID1A) in nccRCC were both demonstrated by large panels and small panels, other less frequent mutations such as MLH3, KMT2D, and CREBBP were not shown by the small panel.

**Conclusion:**

Our study revealed that nccRCC is more heterogeneous than ccRCC. For nccRCC patients, the small panel shows a more clear profile of genetic characteristics by replacing MLH3, KMT2D, and CREBBP with ATM, MSH6, BRAF, and KRAS, which may help predict prognosis and make clinical decisions.

## 1 Introduction

In 2020, 4.3 million patients were diagnosed with kidney cancer, accounting for 1.79 million deaths worldwide (1). There were 75800 newly diagnosed kidney cancer cases and 27800 patients who died of kidney cancer in China (2). Renal cell carcinoma (RCC) is the most common renal tumor in adults, including clear cell RCC (ccRCC), type 1 and type 2 papillary RCC (pRCC), chromophobe carcinoma, and other rare RCCs. ccRCC is the most common subtype, accounting for 75–85% of all cases. Early-stage renal cell carcinoma can be cured by surgical resection. However, recurrent, unresectable, and metastatic RCCs (mRCCs) have a high mortality rate, with a 5-year survival rate of only 12% (3). With the development of targeted therapy and immunotherapy, mRCC survival has been significantly prolonged; however, cancer progression and resistance to therapy need to be resolved, and comprehensive genomic profiles are important for RCC management.

Previous genetic characterization of RCC has significantly increased our knowledge of tumor biology and disease progression. The Cancer Genome Atlas (TCGA) accrued flash-frozen samples of tumor resections and adjacent normal kidneys (or an aliquot of blood if no normal kidney was available) for whole exome sequencing and analyzed the genomic information and related clinical and pathological patient data (4). This project revealed that ccRCC had a specific deletion on chromosome 3 in approximately 90% of patients and most ccRCCs harbored VHL gene mutations. Besides the 3p deletion, TCGA analysis confirmed a frequent occurrence in chromosome 5 (67%) and chromosome 14q (45%) deletions, and the top ten mutated genes in ccRCC were VHL, PBRM1, BAP1, SETD2, KDM5C, TP53, mTOR, SMARCA, PTEN, and ARID1A (5, 6). Numerous epigenomic-related genes are mutated in ccRCC, suggesting that epigenetic regulation plays an important role in the molecular pathways underlying ccRCC leading to the development of possible epigenetic therapies. pRCC is a heterogeneous RCC subtype in which the unifying feature is the presence of papillae in the tumor, which is most commonly separated into type 1 pRCC that has basophilic cytoplasm and type 2 pRCC that has abundant eosinophilic cytoplasm. Genomic profiles have also been described in TCGA studies. Type 1 pRCC is associated with frequent concurrent gains in chromosomes 7 and 17, and numerous potential oncogenes are encoded on chromosome 7, including MET, EGFR, and BRAF. Type 2 papillary RCC was the only loss of chromosome 22 that occurred consistently as a specific copy number alteration (frequency, 30.4%) (7, 8). Compared with type 1 pRCC, type 2 pRCC had low-frequency mutations, and the FH gene (encoding fumarate hydratase) germline and/or somatic mutations were discovered in type 2 pRCC. TCGA has characterized somatic genetic and genomic alterations in RCC; however, these databases are based on Western patients, and only 1.8% of Asian patients were included. Therefore, it is necessary to elucidate Chinese RCC genomic symbols and clinical characteristics of Chinese RCC.

We enrolled 55 patients with RCC from multiple hospitals and performed a panel of 1123 genes sequence, focusing on 79 RCC cancer-related gene target sequences. This study aimed to describe the genomic map of Chinese renal cell cancer and explore the differences between ccRCC and nccRCC, achieving precision medicine for RCC.

## 2 Methods

### 2.1 Patients

Patients were enrolled in three hospitals between November 1, 2021, and August 31, 2022. The pathologist confirmed the diagnosis of renal cell cancer, including ccRCC and pRCC. All participants provided signed informed consent. The specimens used were formalin fixed paraffin-embedded (FFPE) and fresh tumor specimens and were tested by DNA NGS. Clinical demographic parameters, cancer stage using the American Joint Committee on Cancer guidelines, and pathological data including tumor stage and lymph node status were collected.

### 2.2 Next-generation sequence

Tumor samples were collected, and next-generation sequencing tests of all samples were performed at ChosenMed Technology (Beijing) Co., Ltd., Beijing, China). Genomic DNA extraction and library preparation with TruSight™ Oncology 500 (TSO 500) Library Preparation Kit (Illumina, San Diego, CA, United States) were performed following the manufacturer’s protocols. The library was sequenced on an Illumina NextSeq 550Dx platform with a paired-end run of 150 base pairs. Sequence alignment to the human genome (hg19) (9) was completed using the BWA-MEM (version 0.7.11) alignment algorithm. SAMtools (version 1.3) (10) was used to perform the bam-sam conversions. We used the Genome Analysis Toolkit (GATK, version 3.6) (11) module IndelRealigner to perform local realignment of indels. Germline variants were filtered using an in-house built database, and all parameters were set according to the standard protocol (12). Copy number variants (CNVs), including amplification and deletion, were identified using CRAFT copy-number callers from the TSO500 pipeline. Manta (version 1.6.0) (13) was employed to detect large-scale structural variations (SVs) in the RNA library, and only fusions with at least three unique supporting reads, one of which is a split read crossing the fusion breakpoint, were considered candidate fusions. The process of SNVs and indel mutation calling, TMB measurement, and read filtering was performed as described in a previous study. Germline variants were filtered using an in-house built database, and all parameters were set according to the previous workflow. We finally obtained two R packets with 1,123 genes named ChosenOne® and 79 genes named ChsenFocus®.

### 2.3 Statistical analysis

The assessment of clinical characteristics between different cohorts, including age, sex, histological subtype, location, and TNM stage, was performed using SPSS 20.0. The R package “maftools package” (Mayakonda et al., 2018) was applied to perform the mutation analysis and provide a visualized process of variant analysis results. All statistical analyses were performed using R version 3.6.3. All the p-values presented are for a two-tailed test, and p <0.05 represents statistical significance.

## 3 Results

### 3.1 Patients summary

A total of 55 patients diagnosed with renal cell cancer were enrolled from the First Affiliated Hospital of Anhui Medical University, the First Affiliated Hospital of Wenzhou Medical University, and the General Hospital of Eastern Theater Command between November 1, 2021, and August 31, 2022. Among the 55 patients, 78.2% were men and 21.8% were women, with a median age of 57 years. Approximately 69.1% of the patients had ccRCC and 30.9% had nccRCC, including eight with type 2 pRCC and two with type 1 pRCC. Of the tumors, 40.0% were localized to the left kidney, and 58.2% were located on the right side. Of the patients, 52.7% were diagnosed with TNM stage I, and 10% had distant metastases (**Table 1**; **Table S1**).

**Table 1:** Clinical characteristics of 55 RCCs.

Age, median (range)	57 (10~79)
Sex, n (%)	
Men	43 (78.2%)
Women	12 (21.8%)
Histological subtype, n (%)	
ccRCC	38 (69.1%)
nccRCC	17 (30.9%)
Tumor location, n (%)	
Right	22 (40.0%)
Left	32 (58.2%)
Unknown	1(1.8%)
TNM, n (%)	
I	29 (52.7%)
II	5 (9.1%)
III	10 (18.2%)
IV	11 (10.0%)

### 3.2 Somatic mutation of RCC in 1123 gene panel

**Figure 1:**
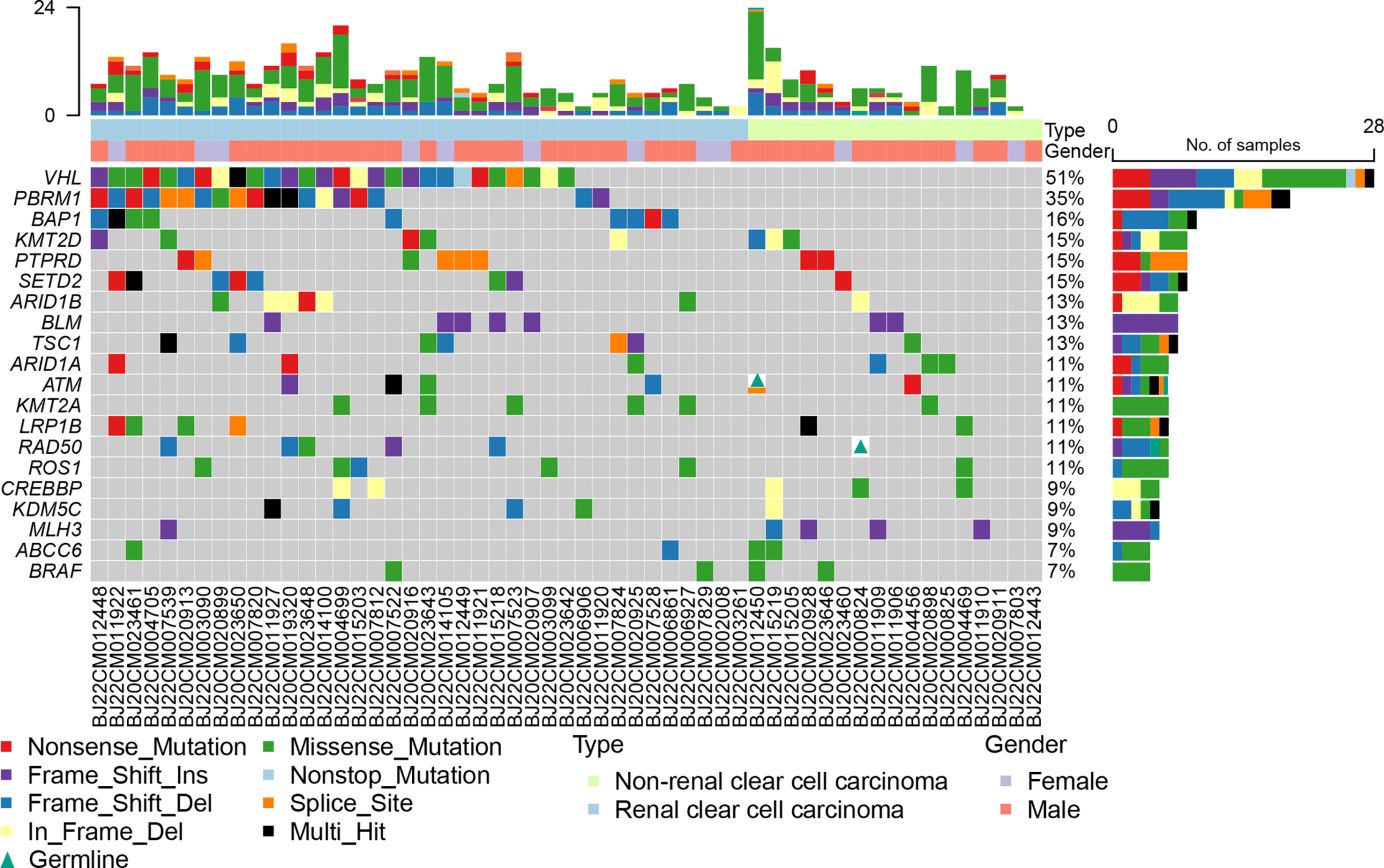
Genomic landscape of 55 RCC patients with 1123 gene.

All the samples were sequenced in an 1123 gene panel. VHL (51%), PBRM1 (35%), BAP1 (16%), KMT2D (15%), PTPRD (15%), and SETD2 (15%) were the most common mutations in all RCC patients **(** **Figure 1** **)**. The mutation frequency in ccRCC was higher than that in nccRCC. Common gene mutations in ccRCC patients were VHL (74%), PBRM1(50%), BAP1(24%), SETD2 (18%), and ARID1B (16%) (**Figure S1**). The mutation copies were lower in nccRCC than in ccRCC, and the most frequent mutations in nccRCC were MLH3(24%), ARID1B (18%), CREBBP (18%), and KMT2D (18%) (**Figure S2**). Missense mutations accounted for the most prevalent mutation in ccRCC, while the most common genetic variation in nccRCC was Fram_Shift. Furthermore, Frame Shift Del and Frame Shift Ins have higher rates of mutation in nccRCC. Specifically, missense mutations in KRAS, NKX2-1, BRAF, CUL3, PRSS1, ABCC6, CYLD, ANKRD11, and BLM only have Frame Shift Ins, whereas BCL10 and MSH6 only have frameshift delay. KMT2D had the highest mutation rate in all three groups when the results of the three groups were examined, and the mutation results of ccRCC were equivalent to those of all RCC patients.

### 3.3 Somatic mutation of RCC in 79 gene small panel

**Figure 2:**
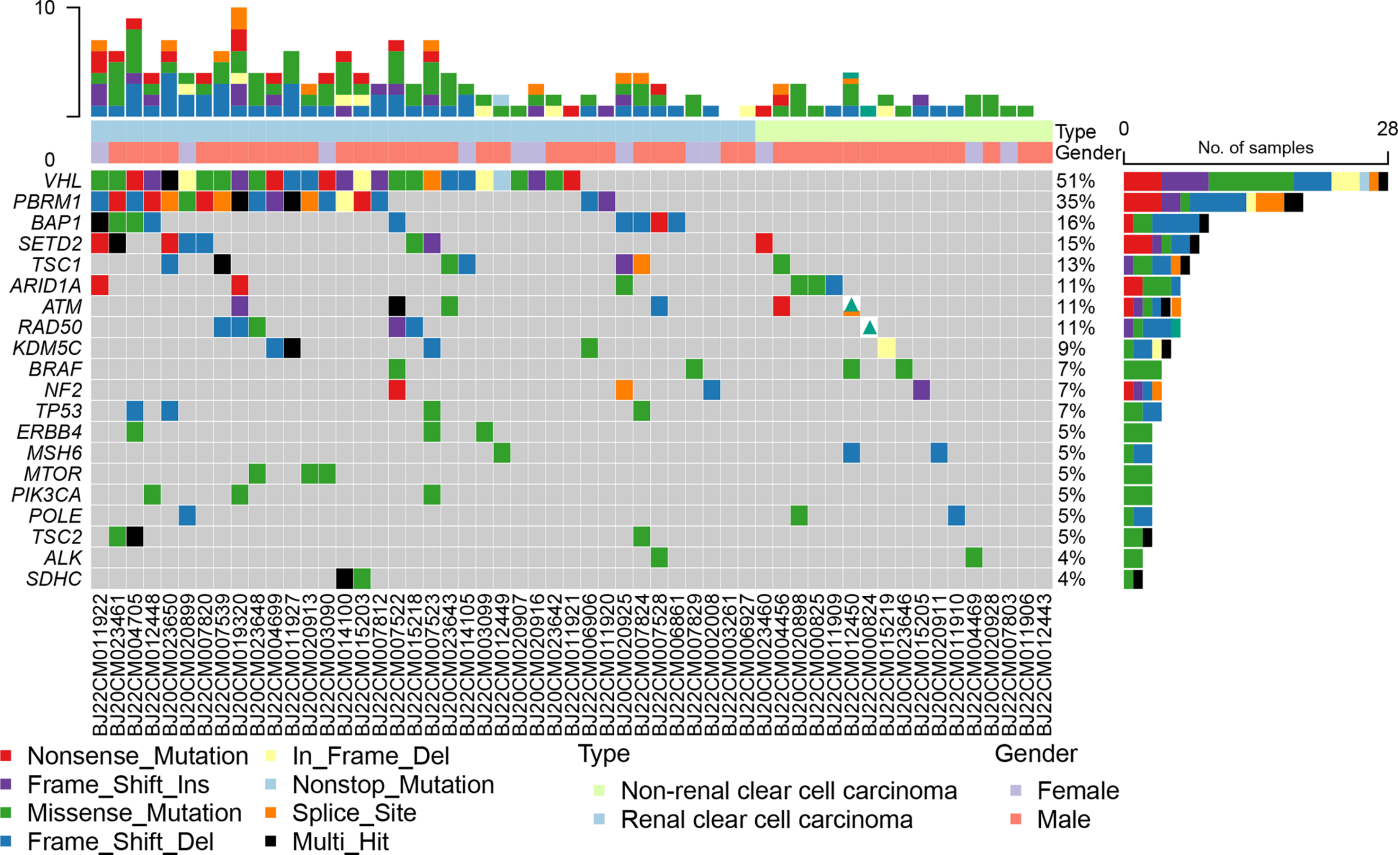
Genomic landscape of 55 RCC patients with 79 gene.

Based on an analysis of 79 genes associated with renal cancer, we found that VHL, PBRM1, BAP1, SETD2, and TSC1 mutation rates were higher in all RCCs at 51%, 35%, 16%, 15%, and 13%, respectively (**Figure 2**). VHL (74%), PBRM1 (50%), BAP1 (24%), SETD2 (18%), and TSC1 (16%) were the most frequently mutated genes in ccRCC (**Figure S3**). nccRCC mutations are highly specific, with high rates of mutations in FH, ARID1A, ATM, BRAF, and KRAS. nccRCC was more heterogeneous than ccRCC (**Figure S4**). The most common type of mutation in both groups of patients was missense mutation, and many genes had only missense mutations. Splice site, Frame Shift Del, Nonsense Mutation, and In Frame Del have all shown independent mutations in nccRCC patients. It seems that ccRCC has a clear driver gene mutation, and patients with ccRCC have a higher mutation rate than those with nccRCC. For ccRCC patients, the mutation profiles in the 1123 gene panel and 79 gene panels were nearly identified, whereas for nccRCC patients, the mutation profiles showed some differences. The most frequent mutations (FH and ARID1A) in nccRCC were both demonstrated by the 1123 gene panel and 79 gene panel; other less frequent mutations such as MLH3, KMT2D, and CREBBP were not detected in the 79 gene panel.

### 3.4 germline mutation of RCC

In 55 patients, we discovered six germline mutations in five (5/55, 9.1%) patients, including four FH genes, one ATM gene, and one RAD50 gene (**Figure S4**); it’s important to note that all six of these germline mutations were discovered in nccRCC (5/17, 29.4%), and no germline mutations were discovered in ccRCC. Four of the five germline mutation patients were diagnosed with type 2 pRCC, three with FH germline mutations, and one with FH mutation concurrent with ATM germline mutation. Patients with a TFE3 fusion have a RAD50 germline mutation.

## 4 Discussion

Since kidney cancer is the most common cancer in urology, we report a comprehensive genomic analysis of 55 RCCs including 38 ccRCCs and 17 nccRCCs to reveal the genomic characteristics of a small Chinese RCC cohort. We discovered that the VHL gene is the most frequent mutation in ccRCC, which was similar to the conclusion that the VHL mutation is the most common mutation of ccRCC according to the TCGA project. Some Chinese researchers have reported that approximately 50% of ccRCC patients have VHL mutations (14), and our results show that VHL is approximately 51% in all RCCs and 78% in ccRCCs, which is similar to that in previous reports. VHL is a key component of the VHL E3 ubiquitin ligase complex that recognizes and binds hydroxylated target proteins in an oxygen-dependent manner. Loss of VHL stabilizes the protein levels of hypoxia-inducible factors HIF1α and HIF2α, which results in a loss of oxygen sensing, induces cellular proliferation, and promotes angiogenesis (15). Besides, VHL, PBRM1, BAP1, and SETD2 are regarded as driver mutations in ccRCC, which also act as biomarkers for ccRCC treatment and prognosis. The PBRM1 gene codes for BAF180, a subunit of the PBAF subtype of the SWI/SNF chromatin remodeling complex, and the PBAF complex suppress the hypoxic transcriptional signature. A study has reported that loss-of-function mutations in the PBRM1 gene were associated with the clinical benefit of using PD-1 inhibitor because PBAF loss shows that RCC is more sensitive to T-cell-mediated cytotoxicity than its PBAF-intact counterparts. Some clinical trials have shown that PBRM1 is a biomarker for immunotherapy (16, 17), but the results are still controversial. Some researchers have reported that PBRM1 loss defines a non-immunogenic tumor phenotype associated with checkpoint inhibitor resistance in renal carcinoma (17). Therefore, more evidence is required to reveal the relationship between PBRM1 mutation and immunotherapy response. In our study, mutations in VHL, PBRM1, BAP1, and SERD2 can reach 74%, 50%, 24%, and 18%, respectively, for ccRCC patients; while for nccRCC patients, the most frequent mutation was FH (29%), MLH3 (24%), ARID1A (18%), KMT2D (18%) and CREBBP (18%). As we know, the inactivation of the Von Hippel–Lindau (VHL) gene is by far the most common oncogenic driver event in ccRCC. Gene mutations in RCC patients were revealed by next-generation sequencing techniques, and the altered genes were then utilized to predict patients’ prognosis and develop therapeutic drugs. The molecular fingerprints described by next-generation sequencing techniques categorize ccRCC into different subtypes that are clinically and therapeutically important. Specific mutations that seem to influence immune cell populations can be discovered in ccRCC tumors because of the interaction between these subtypes and the tumor microenvironment. Opportunities for illness prevention, early identification, prognosis, and therapy have been presented in these studies (18). PBRAM1, BAP1, and SETD2 are chromatin-remodeling genes that are present in the commonly lost region of chromosome arm 3p, which is critical for chromosome stability and remodeling. A lot of studies have revealed that the mutation of BAP1 is associated with poor prognosis (19, 20) even though how PBRM1 gene mutations promote carcinogenesis and tumor progression is still unknown. PBRM1 is considered a tumor suppressor gene by *in vitro* experiments in ccRCC-derived cell lines, which show that PBRM1 gene silencing results in increased proliferation, migration, and colony formation (21).

Joseph RW et al. found that the loss of PBRM1 expression in 1330 ccRCC tumor samples was associated with an increased risk of metastasis without affecting the overall survival (22). The gene mutation of FH was the driving cause of hereditary leiomyomatosis and renal cell carcinoma (HLRCC). The median relapse-free survival for patients with FH gene mutation was only 9 months, so the 2022 WHO classification of renal cell carcinoma has changed the term from HLRCC to FH-deficient RCC which represents a new subtype in nccRCC. FH gene mutation of RCC was the golden standard for FH-deficient RCC, which requires more active treatment.

NccRCC is a rare subtype of RCC, accounting for 15–20% of RCCs, and it is a heterogeneous disease that comprises various types of renal cancer. We recruited 17 nccRCCs to perform the next-generation sequencing techniques, and the results showed that nccRCC has distinct genomic characteristics compared to ccRCC. There were no major mutated genes in nccRCC, and the highest mutated genes were MLH3(24%), ARID1B (18%), CREBBP (18%), and KMT2D (18%), which were lower than those in ccRCC. Numerous potential oncogenes of type 1 pRCC have been reported, including MET, EGFR, and BRAF, and somatic or germline activating mutations of MET has been found in a subset of type 1 pRCC; however, our study did not observe MET mutations due to the small sample size. Nevertheless, we found a higher frequency of FH mutations in type 2 pRCC, which is consistent with a previous report. Some research found that Cabozantinib plus nivolumab is effective in most non-clear cell variants of RCCS, especially those with prominent papillary features, but limited in chromophobe RCCS (23). Over the past two decades, a variety of options have been recognized as the dominant treatment for metastatic renal cell carcinoma (mRCC), including angiogenesis inhibitors, vascular endothelial growth factor receptor inhibitors, other tyrosine kinase inhibitors (TKIs), as well as MET inhibitors and mammalian targeted rapamycin (mTOR) inhibitors. More recently, immunotherapy or combination targeting agents have been shown to significantly improve outcomes in patients with mRCC compared to TKI alone (24).

For all solid tumor gene tests, an 1123 gene panel was designed; however, some genes were not frequently mutated in RCC. We searched for literature and clinical trials and then constructed a panel of 79 genes that were significantly associated with RCC tumorigenesis. Compared to the COSMIC and TCGA databases, the mutation of ccRCC by 79 gene panels is more consistent with the RCC driver mutation. For example, BLM and LRP1B are not significantly associated with the prognosis of ccRCC, but in the 1123 panel, we observed that the frequency exceeded 10%, so the 79 gene panel may be more suitable for profiling RCC gene mutation.

Kidney cancer is an inherited cancer. Several well-known hereditary RCC syndromes account for 5-9% of all RCC cases, including VHL disease, BHD syndrome, and HLRCC. Patients with a family history of RCC have an approximated two-fold increased risk of RCC. Early onset RCC diagnosed before the age of 46 years was reported to be associated with hereditary RCC. In a study of 190 Chinese patients under the age of 45 years who presented with renal tumors, 9.5% had a pathogenic/likely pathogenic (P/LP) germline mutation (25). Our study of 55 RCC patients revealed six germline mutations in five patients (5/55, 9.1%), which was consistent with previous reports. Interestingly, all germline mutations were found in nccRCC, indicating that nccRCC is associated with a high risk of hereditary diseases. We enrolled only seven cases of type 2 pRCC; surprisingly, four of them had FH pathogenic/likely pathogenic germline mutations and one had FH somatic loss. This could be higher than that reported in previous studies of the pRCC germline. FH-deficient RCC is a new WHO 2022 category with more aggressive habits and poor prognosis. A large study cohort including 77 FH-deficient RCC patients observed in the real world has been reported in China (26), with a median progression time of only 21 months, among which 70 patients were confirmed with FH germline mutation and the other 7 patients confirmed with somatic mutation. Therefore, it is necessary to test for germline mutations in nccRCC patients. Furthermore, we found two DDR genes (ATM and BRIP1) germline mutations. Although the DDR germline mutation is not an inherited gene of RCC, some publications have reported DDR germline mutations in kidney cancer in approximately 5% of cases (27, 28); however, the clinical and biological aspects of DDR germline kidney cancer are unknown. There are also differences in genetic mutations between Chinese and Western populations due to ethnic differences. Researchers have found that the five genes with the most mutations in the Chinese population are TP53, KRAS, ARID1A, PBRM1, and SMAD4, while the five most mutated genes in western populations were IDH1, ARID1A, BAP1, TP53, and KRAS. VHL (59.7%), PBRM1 (18.0%), SETD2 (12.2%), BAP1 (10.2%), and TP53 (9.4%) were the most common somatic cell alteration sites in our study. Compared with the TCGA database, the mutation frequency of VHL (59.7% vs. 50.0%, p< 0.001) and TP53 (9.4% vs. 3.5%, p <0.001) in our cohort were higher, while the mutation frequency of PBRM1 was lower (18.0% vs. 31.0%, p < 0.001) in the Chinese cohort (14). Therefore, we believe that racial disparities influence the emergence and progression of RCC. Thus, clinicians would greatly benefit from our work in the prognosis and clinical treatment counseling for RCC in the Chinese population.

Our results described the genomic characteristics of Chinese RCC, revealing that nccRCC has a higher frequency of germline mutations. However, our study had some limitations. First, the study’s limited sample size of Chinese participants raises the possibility that not all RCC genomic alterations are present. This is because, in general, we only performed genetic testing on patients who have reached stage 3 or above. Moreover, genetic testing is still inaccessible for most patients as a result of the price, and some patients cannot afford the entire process. To further enhance our study, we will continue to gather sequencing information from kidney cancer patients in the follow-up study. Second, the gene panel of 1123 and 79 genes could not avoid selection bias. Finally, the mean follow-up time was not long enough; we did not explore the relationship between gene mutations and recurrence.

In conclusion, the present study described commonly mutated genes associated with RCC in a small Chinese cohort and revealed that nccRCC was more heterogeneous than ccRCC, which may help to predict the prognosis and make clinical decisions.

## Data availability statement

The original contributions presented in the study are included in the article/**Supplementary Material**, further inquiries can be directed to the corresponding authors. We declare that the data and materials in this study will be provided free of charge to scientists for noncommercial purposes.

## Ethics statement

The studies involving human participants were reviewed and approved by Ethics Committee of the First Affiliated Hospital of Anhui Medical University. The patients/participants provided their written informed consent to participate in this study.

## Author contributions

ST, D-DX, ZY, and CL contributed to the concept and design of the study. TS, ZY, YG, and SY participated in the writing, review, and/or modification of the manuscript. CL, SX, and JX provided administrative, technical, or material support. All authors contributed to the article and approved the submitted version.

## Supplementary material

Supplementary Figure 1 Genomic landscape of ccRCC patients with 1123 gene.

Supplementary Figure 2 Genomic landscape of nccRCC patients with 1123 gene.

Supplementary Figure 3 Genomic landscape of ccRCC patients with 79 gene.

Supplementary Figure 4 Genomic landscape of nccRCC patients with 79 gene.

Supplementary Table 1 Clinical characteristics of all the patients.
