# Supplementary figures and images for: Genomic profiles of renal cell carcinoma in a small Chinese cohort
Source: Front Oncol. 2023 Jun 22;13:1095775. doi: 10.3389/fonc.2023.1095775 (PMC10324516; doi:10.3389/fonc.2023.1095775)

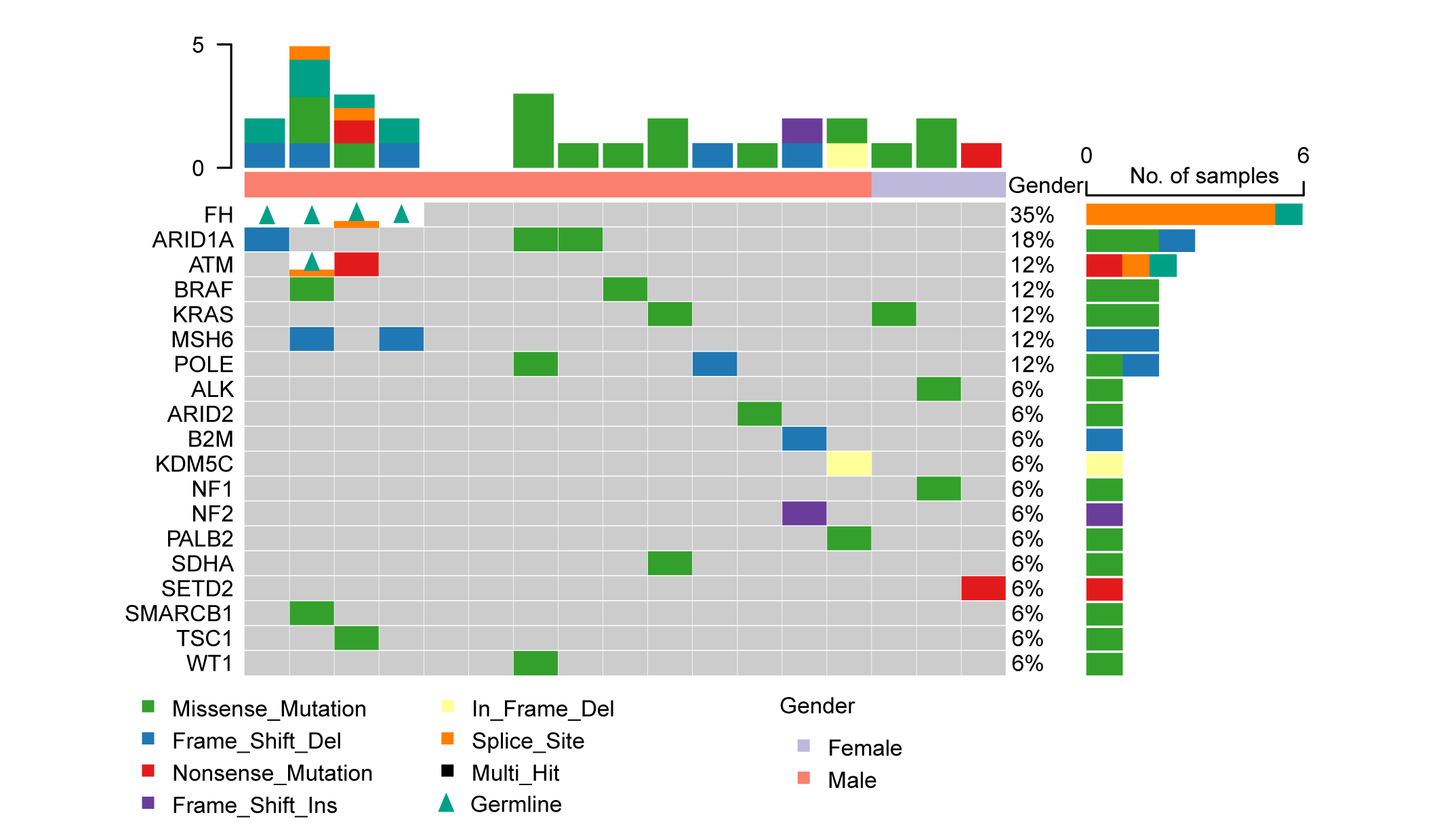

Supplement: Supplementary Figure 1 — Genomic landscape of ccRCC patients with 1123 gene. [file Image_1.tif]

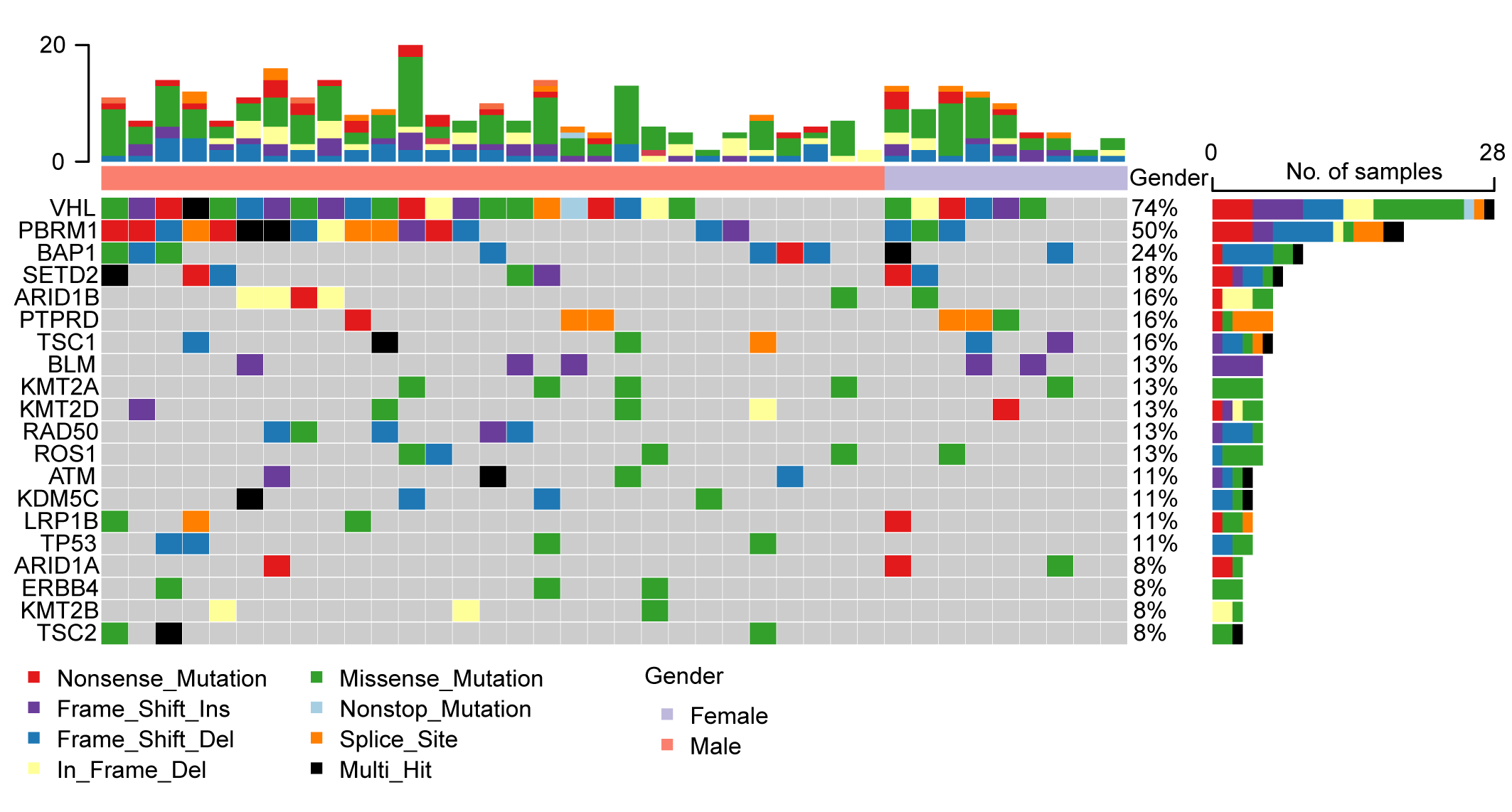

Supplement: Supplementary Figure 2 — Genomic landscape of nccRCC patients with 1123 gene. [file Image_2.tif]

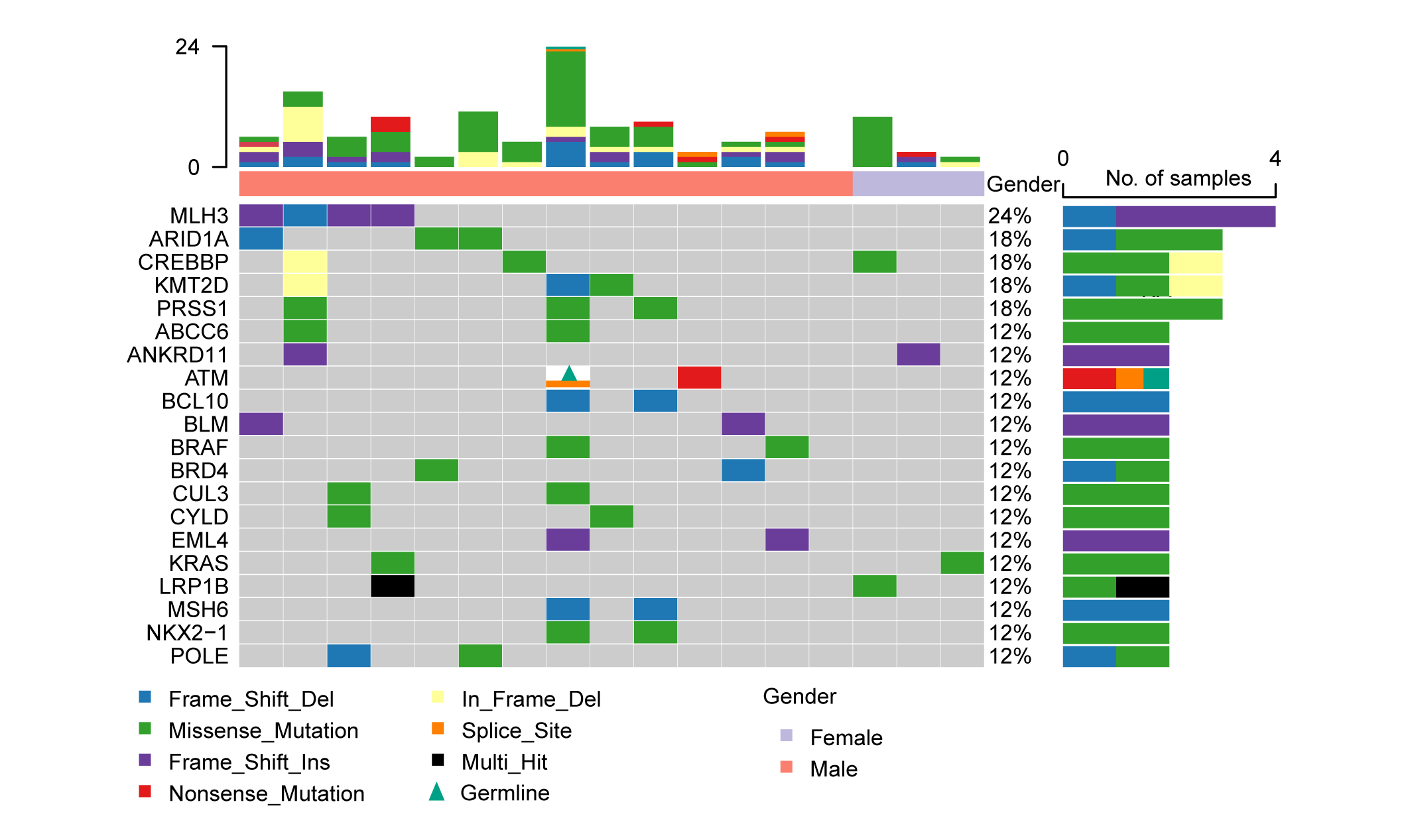

Supplement: Supplementary Figure 3 — Genomic landscape of ccRCC patients with 79 gene. [file Image_3.tif]

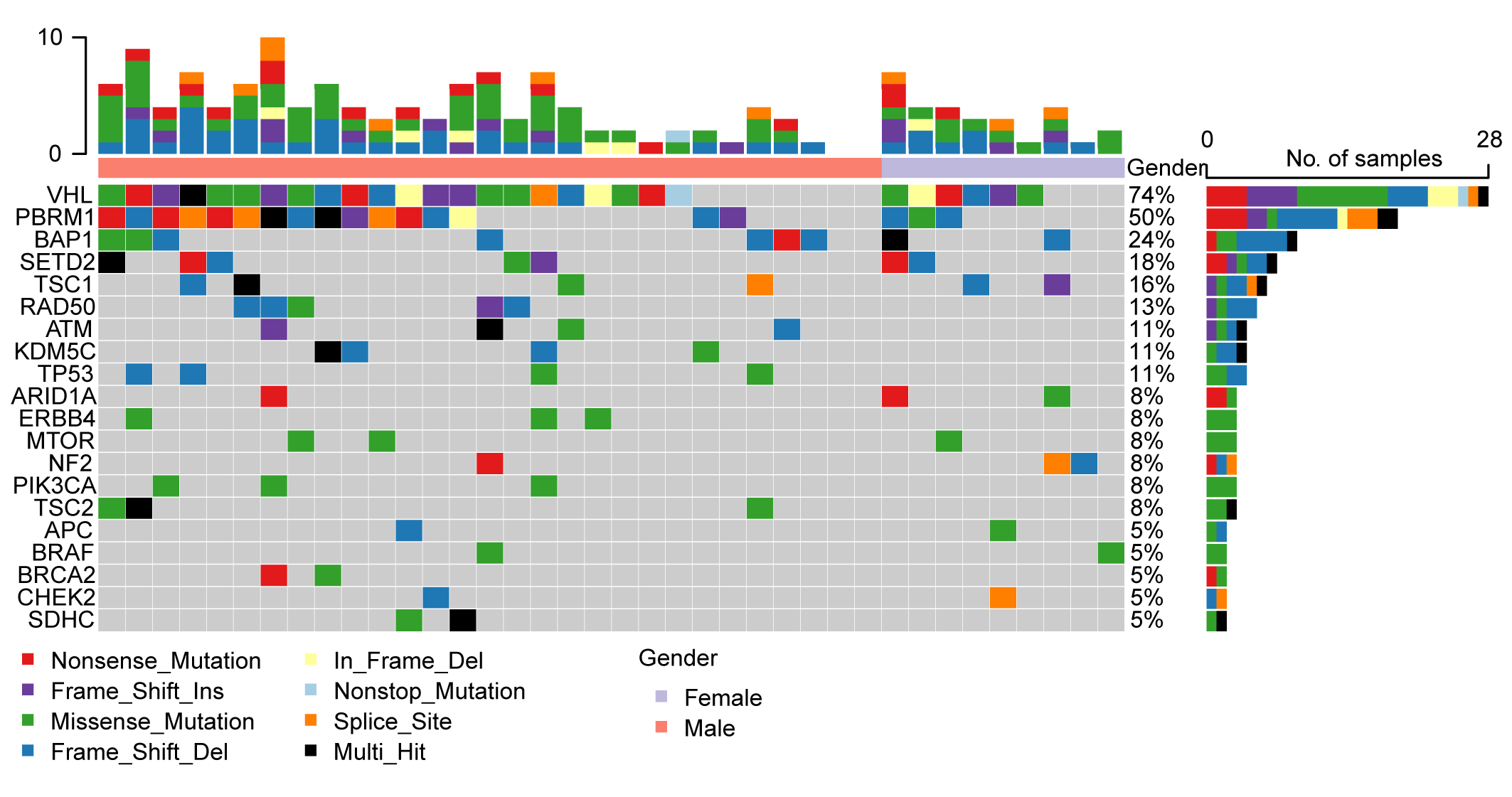

Supplement: Supplementary Figure 4 — Genomic landscape of nccRCC patients with 79 gene. [file Image_4.tif]
